# Occurrence of Methemoglobinemia due to COVID-19: A Case Report

**DOI:** 10.7759/cureus.23155

**Published:** 2022-03-14

**Authors:** Ohoud F Kashari, Salihah A Alsamiri, Fatimah M Zabbani, Dania I Musalli, Ahmed M Ibrahim

**Affiliations:** 1 Pediatric, East Jeddah General Hospital, Jeddah, SAU; 2 University of Medicine and Health Sciences, Royal College of Surgeons in Ireland, Dublin, IRL; 3 Pediatric Endocrinology, East Jeddah General Hospital, Jeddah, SAU; 4 Intensive Care Unit, East Jeddah General Hospital, Jeddah, SAU

**Keywords:** type 1 diabetes mellitus, glucose-6-phosphate dehydrogenase (g6pd) deficiency, atypical covid, methemoglobinemia, covid-19

## Abstract

Methemoglobinemia (MetHb) is a rare, life-threatening condition that occurs when the body is exposed to oxidative stress. It is typically corrected through the glucose-6-phosphate dehydrogenase (G6PD)-dependent shunt. G6PD deficiency is the most common enzymatic deficiency worldwide. This genetic disorder makes patients susceptible to oxidative stress and reduces the expected life span of erythrocytes (red blood cells (RBCs)) among other cells. G6PD deficiency is asymptomatic in most cases unless exogenous stressors are introduced, whether they are dietary, iatrogenic, or infections, such as the highly transmissible serotype of coronavirus, severe acute respiratory syndrome coronavirus 2 (SARS-CoV-2).

We report a case of an 11-year-old male with known insulin-dependent diabetes mellitus (IDDM) and glucose-6-phosphate dehydrogenase (G6PD) deficiency, who was found to develop methemoglobinemia after being infected by the SARS-CoV-2 virus.

The direct effects of COVID-19 on children were reported to be lower than on adults. However, the effects of COVID-19 on children with comorbidities, such as G6PD deficiency in our patient, are understood only to a minimal extent. Moreover, identifying cases of G6PD deficiency prior to initiating treatment with methylene blue, hydroxychloroquine (HCQ), or other contraindicated agents is essential to prevent further deterioration in symptoms.

## Introduction

Methemoglobinemia (MetHb) is a rare, life-threatening condition that can be either congenital, due to enzymatic deficiency, or acquired, due to increased oxidative stress as in the case of exposure to oxidizing agents such as benzocaine. Glucose-6-phosphate dehydrogenase (G6PD) deficiency is the most common inherited enzymatic disorder caused by a genetic defect in the red blood cell (RBC) enzyme, which produces nicotinamide adenine dinucleotide phosphate (NADPH) and protects RBCs from oxidative injury. In G6PD-deficient patients with methemoglobinemia, methylene blue treatment is not recommended since it increases oxidative stress instead of reducing methemoglobinemia (MetHb). Alternatively, MetHb is treated with high-flow oxygen and low-dose vitamin C [1,2].

Since its outbreak in November 2019, the rapid spread of the zoonotic and highly transmissible coronavirus serotype, severe acute respiratory syndrome coronavirus 2 (SARS-CoV-2), has caused severe global disabilities and losses. As of January 2022, there have been around 340,000,000 confirmed cases of COVID-19 and 4,771,408 deaths owing to this pandemic worldwide, including almost 630,000 confirmed cases and 8,914 deaths in the Kingdom of Saudi Arabia [3]. Hospitalization is required in around 7% of COVID-19-positive children who might become critically ill, compared to 25.6% of the adult cases [4].

At the outset of the pandemic, one of the most infamous COVID-19 treatments was proposed that involved the use of hydroxychloroquine (HCQ), which has been proven to be ineffective [5]. Certain case reports, mostly from areas with a low percentage of G6PD-deficient patients, mentioned the occurrence of hemolysis after HCQ treatment was initiated and urged caution when COVID-19 patients present with low hemoglobin [6,7]. However, the association between the two continues to remain uncertain as of the writing of this report [8,9]. A study published by the School of Medicine at the University of Pittsburgh in October 2020 reported no evidence of hemolysis or methemoglobinemia in G6PD-deficient humanized murine model subjected to a high dose of HCQ [10].

A case series published in June 2020 reported three cases of hemolysis and methemoglobinemia in G6PD-deficient patients who received HCQ treatment. The reports proposed that the use of HCQ was not necessarily a leading factor for hemolysis but an exacerbating factor that increases the oxidative stress associated with the illness [11,12].

## Case presentation

Our patient, an 11-year-old male from Saudi Arabia with G6PD deficiency and insulin-dependent diabetes mellitus (IDDM), has been on multiple daily injection (MDI) insulin therapy. This regimen includes long-acting insulin glargine (Lantus) and preprandial rapid-acting insulin aspart (NovoRapid). The total daily dose (TDD) is 27 IU/day (1.17 IU/kg/day) with a basal insulin glargine concentration of 35% and an insulin sensitivity factor (ISF) of 65. Furthermore, neither does he follow any dietary restriction for his diabetes or G6PD deficiency, nor does he correctly apply the insulin dose.

The patient arrived at the polyclinic complaining of a low-grade fever after he had come into close contact with a COVID-19-positive patient. His polymerase chain reaction (PCR) test result was positive. However, since he was stable, he was prescribed paracetamol and discharged with instructions of home quarantine.

After 10 days, he returned to our hospital's emergency department complaining of a three-day history of decreased physical activity, diarrhea, tea-colored urine, skin discoloration, and vomiting, along with a subjective fever. No history of missed insulin doses was reported. The PCR test was repeated and found to be positive.

As per the medical records of the patient, he was diagnosed with type 1 diabetes three years ago after being admitted to the hospital owing to hyperglycemia. No record of his glycemic control or recent HbA1c was available in our hospital. At the age of five, after the consumption of fava beans, the patient was diagnosed with G6PD deficiency. He had a history of hospitalization for febrile seizures when he was three years old. At the time of admission, his immunization was noted to be up-to-date, and he had no surgical history record.

When the patient arrived at the hospital, although he was conscious, he appeared ill and pale. On room air, he had a blood pressure of 113/60 mmHg, a heart rate of 151, and an oxygen saturation level of 69%, with no cyanosis. His random blood glucose (RBG) level was 517 mg/dL (normal range: 90-180 mg/dL). The rest of the physical examination was unremarkable.

On admission, he was administered one bolus of normal saline and was kept on oxygen from a mask at 15 L/minute, raising his oxygen saturation to 79%. Owing to the presence of G6PD deficiency and although the parents denied exposure to fava bean or other relevant triggers, the laboratory results showed significant hemolysis, and he was admitted to the hospital. A urine dipstick test revealed the presence of +4 glucose, +4 ketones, and +4 RBC. A venous blood gas sample evaluation revealed the following: pH = 7.41, CO_2_ = 21.6, and HCO_3_ = 16.3. In the ward, he still required 15 L of oxygen with a saturation of 78%-80%. Based on the test results (Table 1), the patient was found to have an elevated MetHb level of 14.1% (0%-1.5%). Hence, he was shifted to an isolation room in the pediatric intensive care unit (PICU).

**Table 1:** Test results RDW-CV: coefficient of variation of red cell volume distribution width

Test components	Reference range (units)	Admission	Day 1	Day 2	Day 3	Day 4
White blood cell count	6–16 (×10^3^/uL)	19.10	21.90	18.20	22.20	11.10
Neutrophil count	2–8 (×10^3^/uL)	14.50	13.10	11	14.10	6.04
Neutrophil %	45–65 (%)	75.80	59.80	60.60	63.70	54.60
Immunoglobulin	0 (×10^3^/uL)		0.58			0.05
immunoglobulin %	0 (%)		2.64			0.47
Lymphocyte count	1–5 (×10^3^/uL )	3.06	5.75	5	5.34	3.66
Lymphocyte %	25–45 (%)	16	26.20	27.50	24.10	33.10
Monocyte count	0.1–1.1 (×10^3^/uL)	0.99	2.06	1.18	1.98	1.07
Monocyte %	2–10 (%)	5.17	9.42	6.50	8.92	9.67
Eosinophil count	0.1–1 (×10^3^/uL)	0	0.28	0.30	0.27	0.18
Eosinophil %	1–6 (%)	0.02	1.28	1.65	1.21	1.66
Basophil count	0.01–0.1 (×10^3^​​​​​​​/uL)	0.14	0.15	0.17	0.13	0.05
Basophil %	0–1 (%)	0.72	0.66	0.95	0.58	0.47
Red blood cell count	4–5.2 (×10^6^​​​​​​​/uL)	2.67	3.23	3.79	3.30	2.86
Hemoglobin	11.5–15.5 (g/dL)	7.83	9.92	11.60	10.50	8.97
Hematocrit	37.3–47.3 (%)	21.60	27.20	32.90	30.20	26.10
Mean cell volume	81.4–91.9 (fl)	80.80	84.20	86.90	91.60	91.20
Mean cell hemoglobin	25–33 (pg)	29.30	30.70	30.70	31.70	31.40
Mean corpuscular hemoglobin concentration	31–37 (fl)	36.30	36.50	35.40	34.60	34.40
RDW-CV	11.6–13.8 (%)	21	19.90	16.80	15.10	15.30
Platelet	150–450 (×10^3^​​​​​​​/uL)	395	384	257	320	231
Reticulocyte %	0.2–2 (%)	4.13			10.90	
Alanine aminotransferase (ALT)	5–55 (U/L)	132	119			100
Aspartate aminotransferase (AST)	5–34 (U/L)	271	246			108
Chloride	98–107 (mmol/L)	100	105			103
Lactate dehydrogenase (LDH)	125–243 (U/L)	1354				
Potassium	3.4–4.7 (mmol/L)	5.20	4.90			3.7
Sodium	136–145 (mmol/L)	132	137			137
Magnesium	1.7–2.1 (mmol/L)		1.89			1.74
Calcium	8.8–10.8 (mmol/L)					8.1
Alkaline phosphate	141–460 (U/L)	186				
Direct bilirubin	0–0.50 (mg/dL)	0.80			0.76	
Total bilirubin	0.2–1.2 (mg/dL)	2.71			3.62	
Lactic acid	0.5–2.2 (mmol/L)	1.57				
Albumin	3.8–5.4 (g/dL)		3.50			2.9
Serum creatinine	0.3–0.7 (mg/dL)		0.42			0.25
Blood urea nitrogen	7–16.8 (mg/dL)		18			9

**Figure 1:**
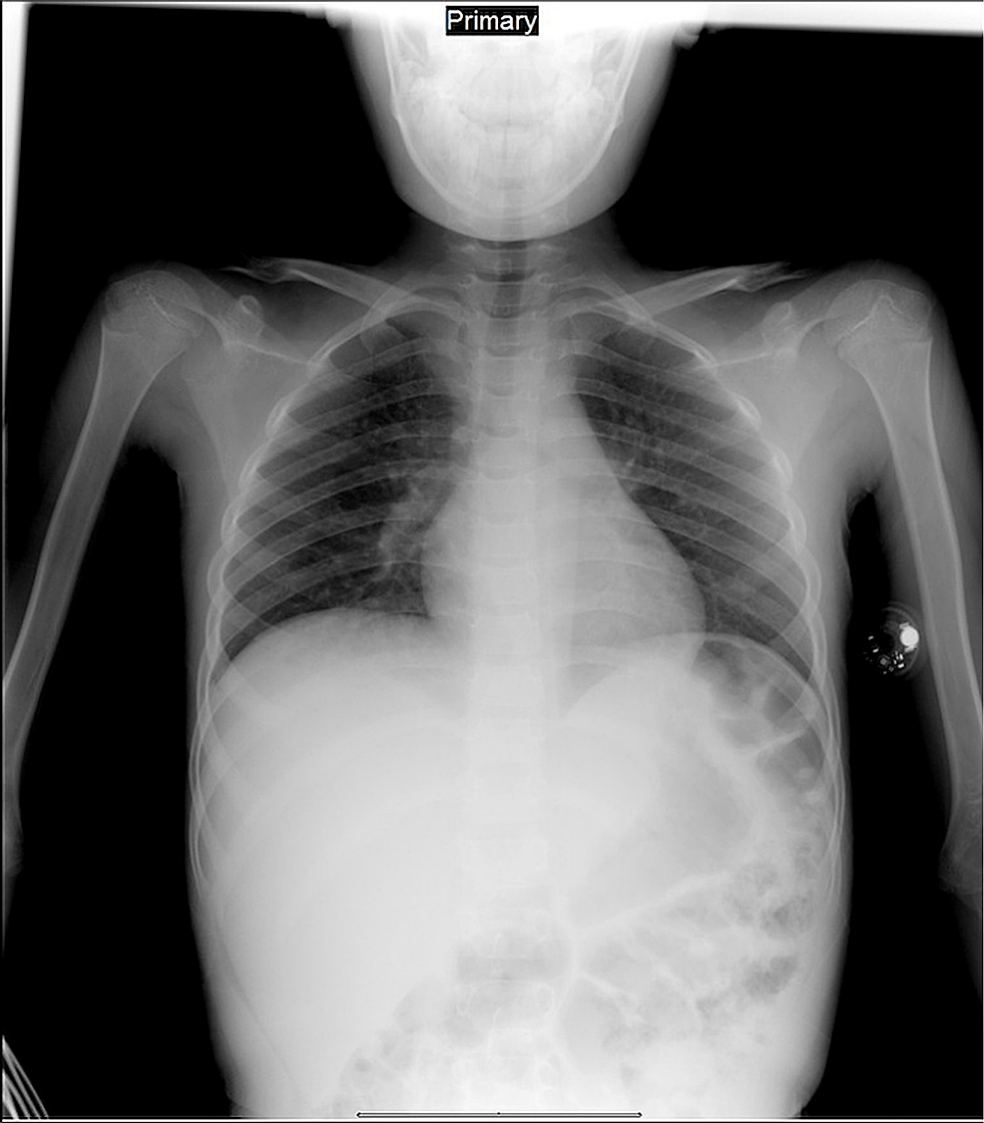
Normal chest X-ray of the patient

His hemoglobin upon admission showed 7.8 g/dL, which necessitated one unit of blood transfusion. The rest of the laboratory results showed signs of hemolysis with high retic and LDH. A chest X-ray was also advised, which was later revealed to be normal (Figure 1).

Upon arrival to the hospital, he received one bolus of normal saline, and due to his low oxygen saturation of 88%, he was on 15 L of oxygen via face mask for 48 hours, and supportive management was continued. Eventually, his oxygen saturation improved to 97%, and thus, it was reduced to 6 L. In addition, his complete blood count and MetHb levels were repeated twice daily, and his vital signs were also closely monitored.

The endocrinology plan aimed to manage the patient's IDDM by achieving an RBS of 90-180 mg/dL.

Initially, the patient continued to take his recommended dose of insulin subcutaneously, monitor his blood sugar level every four hours, and add correction doses of 10%-20% of the TDD. Thus, monitoring and clearing ketosis were achieved with optimal insulin dosing, adequate hydration, and a blood sugar target of 90-180 mg/dL. As a result, the concentration of the intravenous fluid dextrose and subcutaneous insulin requirement was adjusted accordingly.

On the third day, when the patient resumed oral intake, the appropriate regimen for managing his IDDM was adjusted, including 11 IU of Lantus subcutaneously once daily and NovoRapid 5 IU preprandial. Additionally, TDD was set to 1.13 IU/kg/day, 42% basal with ISF 70. Furthermore, the intravenous fluid was stopped.

His urine color normalized on the day of discharge, MetHb levels fell to 1.4% (normal range: 0%-1.5%), and O_2_ saturation was optimal.

## Discussion

G6PD deficiency is an X-linked recessive genetic disorder that affects over 400 million people, making it the most common enzyme deficiency in humans worldwide, and approximately 5% of Saudi Arabia's citizens [13]. This deficiency disturbs the production of nicotinamide adenine dinucleotide phosphate (NADPH), which is the main product of the pentose phosphate pathway and a major electron donor that facilitates multiple anabolic reactions in the body. This genetic disorder makes the patient highly susceptible to oxidative stress and reduces the expected life span of erythrocytes (RBCs) among other cells. In most cases, G6PD deficiency is asymptomatic unless an exogenous stressor is introduced, which could be dietary, iatrogenic, or in the form of infections. These stressors can cause the development of acute hemolytic anemia due to the accelerated destruction of RBCs [14].

Multiple case reports were analyzed that discussed the cases of patients with G6PD deficiency. These patients were treated with HCQ, which led to an elevated oxidative stress status, and eventually, patients developed methemoglobinemia. However, parts of the literature also reported cases about patients who had tested positive for COVID-19 and developed methemoglobinemia without any exposure to HCQ or other oxidative drugs associated with an elevated MetHb in G6PD-deficient patients [15].

The principles of disease transmission emphasize the importance of looking beyond the infectious agent to understand, control, and manage infectious diseases [16]. For G6PD deficiency, the host may play a significant role in the progression of the disease. A 2008 paper discussing the effects of coronavirus serotype 229E showed that G6PD-deficient fibroblasts facilitated a threefold enhancement of viral production compared to control cells. The paper also showed that the G6PD-deficient cells had reduced viability. The research also highlighted how the knockdown of G6PD made epithelial cells more susceptible to death compared to control cells [17]. The epidemiology of SARS-CoV-1 showed reduced symptomatic spread in children, wherein only a small number of children over the age of 12 showed respiratory symptoms. Out of this, approximately 5% needed intensive care unit admission, and less than 1% needed mechanical ventilation. The majority of the children affected by this or any human serotypes of coronavirus showed only mild extra-respiratory symptoms, which mostly involved the gastrointestinal system. The symptoms that were ideally present were diarrhea, abdominal pain, and vomiting, which occurred as a direct result of intestinal invasion by viral particles [18].

The direct effects of COVID-19 infection on children were reported to be relatively lower compared to adults. However, the effects on children with comorbidities, such as G6PD deficiency in our patient, are still only slightly understood. Identifying the presence of G6PD deficiency prior to initiating treatment with methylene blue, HCQ, or other contraindicated agents is essential to prevent further deterioration in symptoms.

## Conclusions

A clinician should be aware of any unusual complications of coronavirus disease caused by the virus or drugs used for its treatment. Methemoglobinemia is a rare but serious medical emergency that must be treated right away. Our patient had several risk factors, including G6PD deficiency, hyperglycemia, and COVID-19 infection. This was sufficient to cause methemoglobinemia. Treating methemoglobinemia in a patient with G6PD deficiency can be complex because methylene blue cannot be used.
